# Time takes space: selective effects of multitasking on concurrent spatial processing

**DOI:** 10.1007/s10339-017-0799-4

**Published:** 2017-03-18

**Authors:** Timo Mäntylä, Valentina Coni, Veit Kubik, Ivo Todorov, Fabio Del Missier

**Affiliations:** 1 Department of Psychology, Stockholm University, Stockholm, Sweden; 2 Department of Life Sciences, University of Trieste, Trieste, Italy

**Keywords:** Multitasking, Spatial relation processing, Cognitive offloading, Time monitoring

## Abstract

Many everyday activities require coordination and monitoring of complex relations of future goals and deadlines. Cognitive offloading may provide an efficient strategy for reducing control demands by representing future goals and deadlines as a pattern of spatial relations. We tested the hypothesis that multiple-task monitoring involves time-to-space transformational processes, and that these spatial effects are selective with greater demands on coordinate (metric) than categorical (nonmetric) spatial relation processing. Participants completed a multitasking session in which they monitored four series of deadlines, running on different time scales, while making concurrent coordinate or categorical spatial judgments. We expected and found that multitasking taxes concurrent coordinate, but not categorical, spatial processing. Furthermore, males showed a better multitasking performance than females. These findings provide novel experimental evidence for the hypothesis that efficient multitasking involves metric relational processing.

## Introduction

Most goal-directed tasks, including multiple-task performance, are temporal in that scheduling, monitoring and task interleaving take place on a time scale, and that coordinating multiple goals and deadlines requires a high degree of cognitive control (e.g., Burgess et al. 2000; Craik and Bialystok 2006; Logie et al. 2011; Redick et al. 2016; Salvucci and Taatgen 2008). We have recently suggested that one way to reduce these executive control demands is to represent the temporal pattern of deadlines and task goals in spatial terms (Mäntylä 2013; Mäntylä and Todorov 2013; Todorov et al. 2014; Todorov et al. 2015). Indirect support for this spatiotemporal view of multitasking is provided by behavioral and neurocognitive studies demonstrating that we understand and handle aspects of time (e.g., duration, sequence) by representing them in a spatial reference frame (for similar views, see, e.g., Bonato et al. 2012; Casasanto and Boroditsky 2008; Dehaene and Brannon 2011). For instance, when we speak about time, we typically apply spatial concepts (e.g., “She has a bright career ahead of her”; see Núñez and Cooperrider 2013). Also, when we respond faster to present information about the past with the left finger and about future information with the right finger, reflecting a spatial left–right mapping of time (Eikmeier et al. 2015).

As a more direct evidence for the spatiotemporal hypothesis of multitasking, we reported studies in which young adults (Mäntylä 2013; Todorov et al. 2015) and older adults (Todorov et al. 2014) with varying spatial abilities completed a multitasking session and separate tasks of executive functioning and spatial ability. Individual differences in executive functioning and spatial ability were shown to be independent predictors of multiple-task monitoring. Furthermore, only spatial ability was related to sex differences in multitasking, and menstrual fluctuation moderated these effects, in that significant sex differences in multitasking performance (and spatial ability) were observed between males and females in the luteal, but not in the menstrual, phase of the cycle.

Extending these findings, Todorov et al. (2015) found that individual differences in multitasking reflect selective effects of spatial ability. Specifically, they relied on the notion that processing of spatial relations reflects two distinct aspects, often referred to as coordinate (metric) and categorical (relative) spatial processing (e.g., Huttenlocher et al. 1991; Kosslyn 1987; Kosslyn et al. 1989; Newcombe and Huttenlocher 2000; also see Laeng et al. 2003, for overviews). Categorical spatial relations refer to equivalent classes of spatial positions relative to a perceptually distinguishable reference object (e.g., left/right, below/above, inside/outside). Coordinate spatial relations refer to more precise spatial locations, which can be expressed in quantitative terms of (e.g., metric distances among spatial elements). Patient studies (e.g., Laeng 1994, 2006; Palermo et al. 2008), experimental findings (Kosslyn 1987; Kosslyn et al. 1989) and brain imaging studies (e.g., Baciu et al. 1999; Kosslyn et al. 1998) support the distinction between categorical and coordinate spatial processing (for overviews see also Laeng et al. 2003; Van der Ham et al. 2014). Importantly, Todorov et al. (2015) hypothesized and found that individual differences in coordinate (but not in categorical) spatial processing contribute to multitasking performance. Individuals who were good at handling metric spatial relations were also better multitaskers than individuals with less efficient coordinate spatial skills. Furthermore, these effects were accentuated by sex-hormone-related fluctuation across the female menstrual cycle. Specifically, sex differences in multitasking and coordinate (but not categorical) spatial processing were observed between males and females in the luteal phase of the menstrual cycle (during which estradiol levels are heightened). By contrast, sex differences in both multitasking and spatial relation processing were eliminated between males and females at menses (during which estradiol levels are reduced).

These findings suggest that sex-related differences in spatial ability are observed in coordinate-type, metric relational processing, but not in more categorical, nonmetric spatial processing. Furthermore, they also suggest that individual (and sex-hormone-related) differences in coordinate, rather than categorical, spatial processing, contribute to temporal coordination of multiple tasks.

An implication of these findings, and consistent with our spatiotemporal hypothesis of multitasking, is that increasing concurrent demands on spatial relation processing should have a larger cost when they involve metric, rather than categorical, spatial relational processing. By contrast, if multitasking performance is not selectively related to differences in spatial relation processing, concurrent coordinate and categorical processing should show comparable secondary-task costs.

In the present study, we examined the implications of the spatiotemporal hypothesis, experimentally testing its predictions by varying concurrent-task demands on metric vs. categorical processing. Participants completed a multitasking session with four identical and simple component tasks, requiring a high degree of coordination among the tasks. In this *counter* task, participants have to monitor four digital “clocks” (counters) that are identical, in that they display forward-running digits, and instructions are to press the spacebar whenever one of the counters shows a target reading, which was defined by a simple rule (see also Mäntylä 2013; Todorov et al. 2014, 2015). Participants completed a baseline multitasking condition (referred to as the no-load condition) and, to manipulate concurrent spatial load, they also carried out multitasking sessions concurrently with separate coordinate and categorical spatial processing tasks (see also Michimata 1997; Palermo et al. 2012; for details, see the “Method” Section).

Following the reasoning outlined above, we expected that coordinate and categorical tasks would show similar levels of performance when completed as single tasks (suggesting that the two tasks are equally difficult). However, when completed in combination with the counter task, we expected larger concurrent costs of multitasking on coordinate than categorical relational processing. We reasoned that these effects would be observed in the spatial task performance, as participants were expected to focus their reduced attentional resources on the counter-task performance (considered as the primary task) at the expense of spatial task performance. As a support for this hypothesis, Todorov et al. (2014) reported a study, in which young and old adults completed a similar monitoring task (with three counters running at different rates) along with a concurrent working memory (n-back) task. Age differences were observed in both counter and n-back task performance, but these differences were about three times larger in the latter task, suggesting that older participants focused their limited resources on the (primary) counter task at the expense of secondary-task performance.

A secondary aim of the study was to examine the generality of our earlier findings showing sex differences in multitasking by involving a less selected group of female participants. As elevated levels of estradiol have been found to increase the magnitude of sex differences in spatial ability (e.g., Hampson et al. 2014; Halpern 2012), we accentuated these hormone-related effects in our previous studies by eliminating females with reduced sex-hormone fluctuation due to, for example, hormonal treatment, use of hormonal contraceptives, or pregnancy. Instead, the present sample of female participants was more representative than in our earlier studies (involving Swedish participants) in that we included females independent of their hormonal status.

## Method

### Participants

A total of 62 University of Trieste undergraduates (37 women) between 19 and 38 years of age (*M* = 22.08, *SD* = 4.01) participated in the study in return for partial course credit. Sample size was determined on the basis of our previous studies with similar tasks. Specifically, in Todorov et al. (2015) study, we found a correlation of .30 between spatial ability (as measured by the mental rotation test, MRT) and the counter-task performance, and a post hoc power analysis showed a good power (.85, two tailed). Based on an estimated minimum correlation of .32 between MRT and counter-task performance, we then estimated the approximate number of participants for the power of .80. An a priori power analysis for this study provided an estimate of N = 60 for a .80 power (two tailed).

### Tasks and procedure

Spatial performance, both in terms of concurrent and baseline performance, was based on the coordinate and categorical versions of the clock-face task (see also Todorov et al. 2015). The two tasks were nominally identical in that participants were presented with digital time-readings (e.g., 07:10) on a computer screen and were instructed to imagine the stimulus time as the hands of an imaginary analog clock. For the *coordinate* task, participants were asked to indicate which of two concurrently presented digital readings (e.g., 13:49–07:10) formed a larger angle between the hour and the minute hands on an imagined analog clock face. To equate task difficulty, the categorical task involved three concurrently presented digital readings (e.g., 13:49–07:10–02:37). Participants imagined the position of the clock hands relative to the four quadrants of an analog clock, and they indicated whether any of the quadrants were “free” (e.g., 13:49 and 07:10 occupy the first, third and fourth quadrants and 02:37 the first and fourth quadrants, respectively, leaving the second quadrant free). None of the stimuli resulted in vertical (6 and 12) or horizontal (3 and 9) hand positions that could be perceived as ambiguous. Both tasks comprised 20 items and the test phase was preceded by a set of practice items, during which an analog clock face (without the clock hands) was displayed as a support. Participants responded by pressing designated keys, and they were instructed to respond as quickly as possible while avoiding mistakes. Response time (*max* = 20 s) and accuracy were the dependent measures of both tasks.

Multitasking was assessed with the counter task. In this time-based monitoring task, four digital clocks, or counters, were occluded by colored rectangles on the computer screen (see also Mäntylä 2013). Participants could monitor each counter by pressing a specific key, whereupon the corresponding counter appeared for 2 s. To prevent the four tasks from being handled as a unitary task, the counters ran at different rates (4.2, 3.7, 2.7, and 2 s per item, respectively). Participants pressed spacebar whenever one of the counters displayed a target reading defined by a simple rule. Participants were instructed to press spacebar when the last digit of the Green Counter (running at 4.2 s/item) was 7, when the last two digits of the Blue Counter were a multiple of 11, when the last two digits of the Red Counter were a multiple of 20, and when the last two digits of the Yellow Counter (running at 2 s/item), were a multiple of 25. Participants could check the reading of each counter whenever they wanted by pressing a designated key on the keyboard.

Participants were tested individually during a single session. Informed consent was obtained before participation, and the study was completed according to the ethical guidelines established by the Declaration of Helsinki. All the tasks were computerized, and the stimuli were presented on a 20″ display. Each task included separate instructions and a practice phase during which the experimenter checked that instructions were properly understood. After a brief questionnaire about demographic background, participants completed the coordinate and categorical tasks in a counter-balanced order, followed by the first counter-task session (without load). During the following concurrent-task session, participants completed the counter task along with the coordinate and categorical spatial tasks in a counter-balanced order.

In these two concurrent tasks, digital times were presented above the four counters at the rate of 20 s per item, and participants reported which angle of two pairs of clock hands was larger (coordinate) or whether three pairs of clocks hands occupied all four quadrants of a clock face (categorical).

Multitasking performance was based on a combined score of the four counter tasks (see also Mäntylä 2013), with response accuracy and monitoring frequency as dependent measures. As the latter measure did not show any systematic effects, accuracy was the primary measure of counter-task performance. A response was considered correct if the spacebar was pressed within one digit of the target (e.g., the digits of 19, 20, and 21 would be considered correct responses if the target was 20). The coordinate and counter-task performance was based on accuracy and response time, and both data are reported here. The data were submitted to two main analyses, first examining the hypothesis that multitasking performance, as measured by the counter task, is reduced by concurrent spatial task (i.e., counter-task performance under spatial load vs. no-load) and, followed by a more specific analysis in which we examined concurrent costs of multitasking on coordinate and categorical relational processing.

## Results

**Fig. 1:**
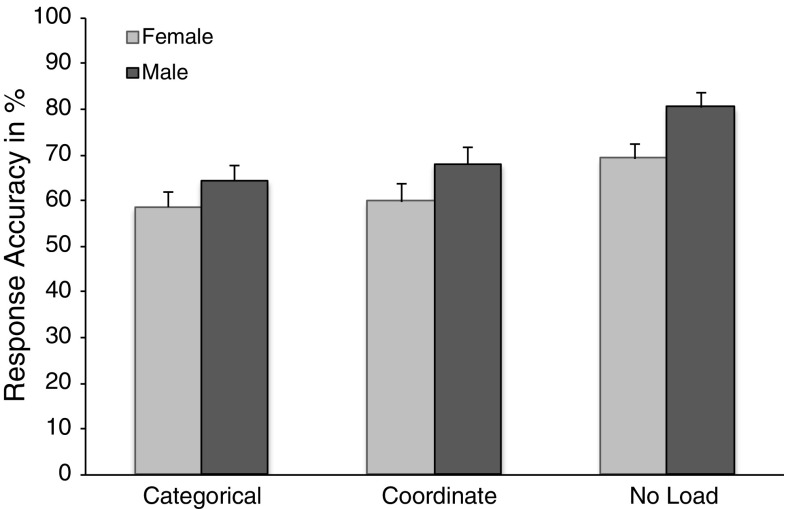
Counter-task accuracy as a function of gender and concurrent task. Error bars indicate standard error

Figure 1 summarizes the outcome of the first analysis on counter-task accuracy as a function of task load and sex. These results suggest that, compared to the no-load condition (*M* = .73, *SD* = .13), concurrent spatial processing reduced counter-task accuracy, *F*(2, 116) = 41.91, *MSe* = 61.39, *p* < .01, with comparable effects for the categorical (*M* = .61, *SD* = .14) and coordinate (*M* = .64, *SD* = .13) conditions. Furthermore, males (*M* = .72, *SD* = .11) outperformed females (*M* = .63, *SD* = .11), *F*(1, 58) = 11.11, *MSe* = 347.76, *p* < .01. No other effects were observed.

**Fig. 2:**
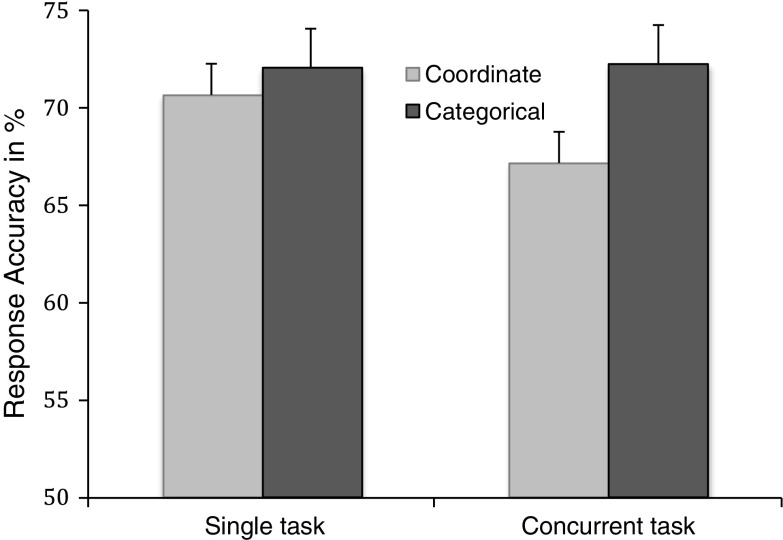
Coordinate and categorical accuracy as a function of task. Error bars indicate standard error

Figure 2 summarizes coordinate and categorical spatial data under the single and concurrent-task conditions. These results show similar levels of performance in the single-task conditions, suggesting that the two spatial tasks were equally demanding. However, when completed in combination with the counter task, coordinate task performance was less accurate than categorical task performance. As shown in Fig. 2, compared to the single-task condition, categorical task performance was not affected by the concurrent-task condition, whereas the coordinate data showed a clear difference between the single- and concurrent-task conditions.

An ANOVA on the accuracy data confirmed these observations by showing a main effect of test session (single vs. concurrent), *F*(1, 59) = 21.18, *MSe* = 6.78, *p* < .01, and a significant session x task interaction, *F*(1, 61) = 24.06, *MSe* = 184.22, *p* < .01. The contrast between the single and concurrent tasks was significant for the coordinate, *t* (61) = 3.51, *p* < .01, but not for the categorical, data. Similarly, the contrast between coordinate and categorical conditions was significant in the concurrent-task condition only, *t*(61) = 2.11, *p* < .05. The main effect of sex and its interactions were nonsignificant.

An ANOVA on the response-time data also showed a main effect of test session, *F*(1, 59) = 79.31, *MSe* = 42,116, *p* < .01, suggesting that participants were under greater time pressure in the concurrent-task condition (*M* = 10.55 s) than in the single-task condition (*M* = 11.99 s). Furthermore, the main effect of task was significant, *F*(1, 59) = 20.89, *MSe* = 96,757, *p* < .01, with longer response times in the categorical condition (*M* = 11.66 s) than in the coordinate condition (*M* = 10.58 s), indicating that spatial relation judgments involving three clock hands were more time consuming than those involving two pairs of clock hands. No other effects were observed.

## Discussion

The starting point of this study was our earlier correlational findings suggesting that individual differences in multitasking performance reflect selective effects of spatial relation processing (Mäntylä 2013; Todorov et al. 2014, 2015). Extending these results, we tested experimentally the hypothesis that concurrent spatial processing should have a larger cost when they involve metric, rather than categorical, spatial relational processing.

Taken together, the results of this study supported our primary hypothesis. First, the baseline data showed that the coordinate and categorical versions of the clock-face task were equally demanding, as measured by accuracy. Yet, only coordinate spatial processing was associated with concurrent-task costs. These costs were observed in spatial task performance, rather than in counter-task performance. This result is consistent with the findings of Todorov et al. (2014) with young and old adults in that older participants attempted to maintain primary task performance at the expense of secondary-task performance (see also Mäntylä et al. 2007, 2009, for similar findings for dual-task performance in children and older adults).

We also found consistent sex effects in multitasking in that males showed better performance than females in counter-task accuracy. This result extends previous findings (Mäntylä 2013, Todorov et al. 2015; but see also Redick et al. 2016; Strayer et al. 2013) by showing sex differences in multitasking in a nonselected group of participants. We accentuated hormone-related effects in our previous studies by eliminating females with reduced sex-hormone fluctuation due to, for example, hormonal treatment, use of hormonal contraceptives, or pregnancy. The present study shows consistent sex differences in multitasking favoring males even when sex-hormone-related effects were not considered.

Sex differences were observed in primary task, but not in secondary-task performance. This result might reflect strategic differences in that both females and males considered the primary task as more important than the secondary spatial task, thereby reducing potential sex differences in spatial task performance. Furthermore, the 20-s response time of both spatial tasks might also been too lenient for sex differences in secondary (coordinate) task performance, especially in a nonselected group of female participants.

A central finding of this study was that concurrent task involving metric (vs. nonmetric) spatial task had selective effects on multitasking performance. This result increases the generality of Kosslyn (1987) and others view that spatial relational processing involves two complementary spatial processes, and that these relation processes are not limited to spatial domains but may also contribute time-related processes and temporal coordination of multiple deadlines.

In more general terms, these findings are consistent with the hypothesis that complex cognitive tasks, such as multitasking or memory for multiple intentions, require high degrees of cognitive control, but that these tasks may also reflect a form of cognitive offloading of executive control demands. In this spatiotemporal offloading hypothesis of multitasking, we suggest that executive control demands involved in temporal coordination of complex patterns of deadlines can be alleviated by transforming temporal relations to spatial relations, and that individuals with efficient (metric) spatial abilities are better multitaskers than individuals with less efficient spatial skills. The findings of this study support this hypothesis by showing selective effects of concurrent metric, but not nonmetric, spatial processing. In other words, multitasking performance was compromised when possibilities for spatiotemporal offloading were reduced in both spatial conditions, and these effects were accentuated when demands on concurrent metric spatial processing were increased in the coordinate task condition.

Although the findings of this study are consistent with our spatiotemporal hypothesis of multitasking, it is important to acknowledge the limitations of their implications. First, a central assumption of our framework, which we have emphasized in earlier work (Mäntylä and Todorov 2013; Todorov et al. 2015), is that expertise and executive control functioning are the primary sources for individual differences in multitasking. Experts are better than nonexperts in handling multiple tasks (e.g., air traffic control, Loukopoulos et al. 2009; Wickens 2008; preparing a breakfast in one’s own kitchen), and individuals with efficient executive functions are typically better multitaskers than individuals with less efficient control functions (Redick et al. 2016; Shallice and Burgess 1991). However, in many novel or unfamiliar situations, overlearned scripts and schematic knowledge structures are not relevant (or even interfering) for handling executively demanding task coordination (cf. preparing a breakfast in someone else’s kitchen). In these conditions, recoding temporal patterns of deadlines to spatial relations may reduce executive control demands of multiple-task coordination.

As the findings of this study suggest, multiple-task performance is reduced when access to this time-in-space offloading is limited. However, a boundary condition of this hypothesis is that multitasking reflects individual differences in spatial ability (including concurrent-task costs) *only* when demands on temporal coordination are high. Thus, the role of spatial ability should be reduced or even eliminated under flexible deadlines and time windows. Similarly, the contribution of spatial skills should be only marginal in dual-task performance, in which demands on temporal coordination are minimized. This hypothesis is also supported by studies in which “multitasking” requires the coordination of two component task (cf. driving while talking to the phone, Mäntylä and Todorov 2013).

Taken together, the present findings provide experimental evidence for our spatiotemporal hypothesis of multitasking, extending previous correlational findings that showed the relevance of spatial processing for multitasking. Furthermore, these effects were selective in that only coordinate-type of metric spatial processing suffered from concurrent multiple-task performance (and not categorical spatial processing), even when the two spatial tasks were equally demanding in terms of single-task performance.

To the extent that coordinate-type of spatial relation processing plays a central role in multiple-task performance, an interesting avenue for future work would be to identify the specific mechanisms of coordinate versus categorical spatial processing and to relate these functions to individual differences in multitasking. Is efficient multitasking associated with the metric (noncategorical) nature of coordinate processing, or is coordinate task performance a proxy for some more basic operations? A related implication of these findings is that training of spatial relation processing might facilitate multitasking performance (Strobach et al. 2012; see also Cardoso-Leite et al. 2015; Uttal et al. 2013), and that these effects might be accentuated in individuals with less efficient skills in spatial relation processing.
